# Successful Re‐Pigmentation of IPL‐Induced Hypopigmentation Using Topical Bimatoprost

**DOI:** 10.1111/jocd.70619

**Published:** 2025-12-24

**Authors:** Ivaylo Ivanov, Tatjana Pavicic

**Affiliations:** ^1^ Private Practice Dr. Tatjana Pavicic Munich Germany

## 1 Case Presentation

A 38‐year‐old healthy, Fitzpatrick phototype IV female patient presented to our clinic for aesthetic treatment of leg vessels. On clinical examination, multiple thin telangiectasia were noticed. Due to the lack of a long‐pulsed Nd:YAG laser and considering the thickness and depth of the vessels, a possible treatment with intense pulsed light (IPL) was considered. After thoroughly discussing limitations and possible risks of performing IPL treatment on Fitzpatrick phototype IV skin with the patient, a small, approximately 8 cm^2^ test spot was performed, using the IPL handpiece (MaxG) of the Icon platform by Cynosure Lutronic (Cynosure Lutronic, Westford, Massachusetts, USA).

Based on the thermal relaxation time of the targeted vessels, a pulse width of 20 ms was selected for the treatment. Initial fluence was set to 44 J/cm^2^, which did not reveal any visible clinical reaction to the vessels or the surrounding skin. After gradually increasing the fluence to 48 J/cm^2^, an immediate gray dyspigmentation accompanied by epidermolysis was observed. The treatment was discontinued and a potent topical clobetasol cream was applied to the treated area. The patient was discharged with a prescription for topical fusidic acid/betamethasone valerate combination cream. Detailed instructions on appropriate wound care and maintaining the area moist with plain petrolatum were provided.

**FIGURE 1:**
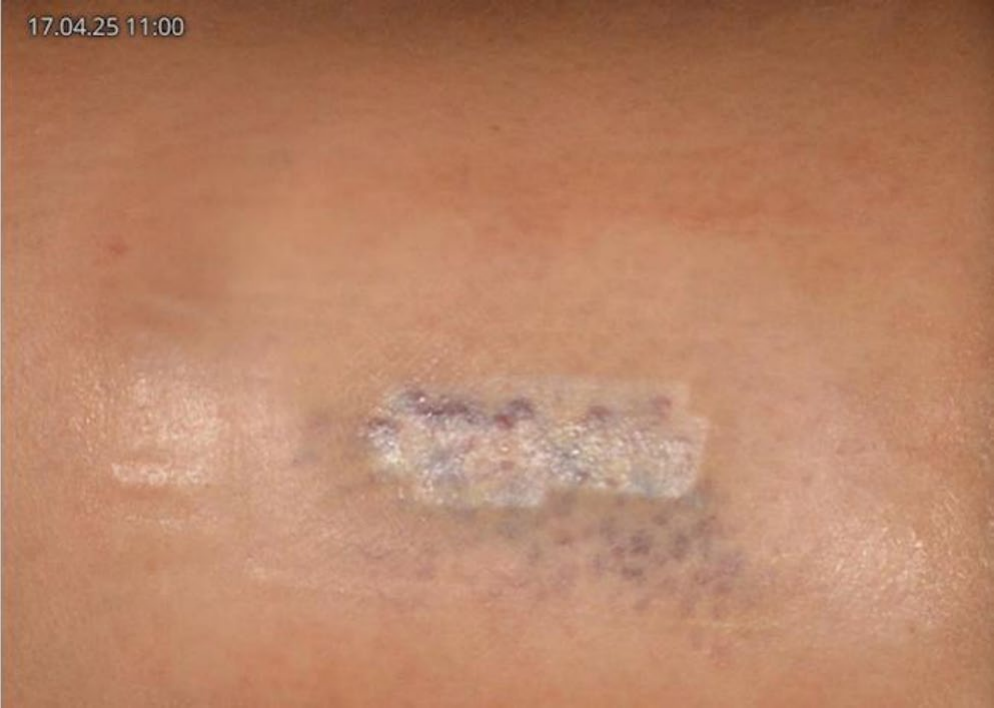
Post‐inflammatory hypopigmentation following IPL burn.

The patient was closely followed up twice weekly. After the acute burn reaction had subsided and the crust had fallen off, a sharply demarcated hypopigmentation corresponding to the spot size of the IPL handpiece was revealed (Figure 1):

The patient was instructed to strictly avoid sun exposure. Furthermore, to mitigate any scar reaction, platelet‐rich plasma (PRP) therapy was initiated on a twice‐weekly basis. Approximately 3 mL of PRP were injected twice weekly for a total of 8 weeks. Fortunately, there were no scar reactions observed and the area healed without any hypo‐ or hypertrophic changes. However, the hypopigmentation persisted. A single 1540 nm fractional non‐ablative laser treatment did not seem to influence the pigmentary aberration.

**FIGURE 2:**
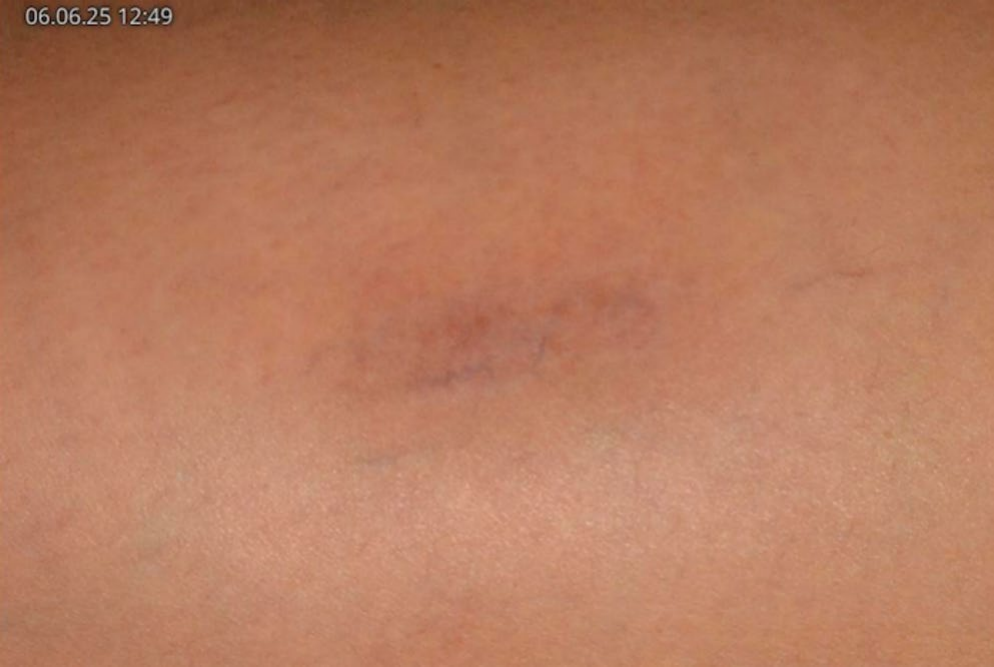
Near‐complete resolution of the IPL‐induced hypopigmentation, 3 weeks after starting bimatoprost.

At the post‐IPL Week 9 follow up session, the patient was informed about possible treatment of the hypopigmentation with topical bimatoprost. Topical bimatoprost ophthalmic solution was prescribed and the patient was instructed to apply the medication on a nightly basis after washing the area. Additionally, the bimatoprost solution was applied once weekly in‐office after puncturing the hypopigmentated surface area several times with a 30 gauge needle until pinpoint bleeding was noticed. Afterwards, petroleum jelly was applied to the wound and sterile dressing was provided. One week after initiating the bimatoprost treatment, the patient stated that she was seeking marked improvement of the hypopigmentation. Clinically, a clear re‐pigmentation of the area was appreciated. The in‐office application of bimatoprost after puncturing the skin was continued for four more weeks. By the end of this period, the hypopigmentation was barely perceptible, with near‐complete restoration of skin tone (Figure 2):

The patient was instructed to apply bimatoprost once weekly and a follow‐up appointment was scheduled for 1 month later.

## 2 Discussion

Pigmentary changes are common adverse effects of laser and intense pulsed light (IPL) treatments. Consideration of treated lesions, skin type and careful selection of device parameters (including observation of laser/IPL‐tissue interactions) are essential aspects of safe and effective treatments. Even with ideal patient and device/settings selection, post‐inflammatory pigmentary alterations can occur.

To the best of our knowledge, this is the first report of successful treatment of IPL‐induced hypopigmentation with topical bimatoprost. Topical prostaglandin analogues such as bimatoprost have been reported to stimulate melanogenesis and may therefore be beneficial in repigmenting hypopigmented scars. Bimatoprost is a synthetic prostaglandin F2α analogue and has been shown to stimulate melanogenesis by increasing tyrosinase activity in melanocytes. It was originally introduced for glaucoma treatment and eyelash growth. Due to its cutaneous hyperpigmentation side effects, the medication has been therapeutically repurposed for hypo‐ and depigmented lesions [1].

Bimatoprost penetration can be enhanced by different methods which temporarily enhance the epidermal permeability, mainly lasers and microneedling [2]. Fractional non‐ablative and ablative laser technologies enable precise dermal remodeling while facilitating laser‐assisted drug delivery (LADD) to enhance therapeutic penetration of pharmacological drugs [3]. Fractional ablative lasers (Erbium:YAG, CO_2_) are generally more effective for drug delivery due to the physical removal of skin; however, they are associated with more downtime and wound healing [4]. Microneedling can serve as a transcutaneous delivery alternative to LADD; although large population studies with direct comparison are currently not available, LADD might be more effective [5].

Post‐inflammatory hypopigmentation is generally most challenging to treat and regarded by many as irreversible. In clinical practice, treatment options are severely limited and include laser resurfacing [6] (e.g., Erbium:Glass 1550 nm laser), UVB irradiation (e.g., 308 nm Excimer laser [7]), and more invasive procedures such as excision and melanocyte transfer [8]. Topical bimatoprost thus may offer a cost‐effective, low‐risk treatment option to patients with laser‐ and IPL‐induced hypopigmentation.

The results of this case report are severely limited by the small sample size, lack of control group and blinding. Thus, the treatment efficacy in the setting of IPL‐induced hypopigmentation could only be confirmed by a great series of successful treatments.

## Conflicts of Interest

The authors declare no conflicts of interest.

## Data Availability

The data that support the findings of this study are available on request from the corresponding author. The data are not publicly available due to privacy or ethical restrictions.

## References

[1] S. Fukaya , M. Kamata , T. Kasanuki , et al., “Open‐Label Pilot Study to Evaluate the Effectiveness of Topical Bimatoprost on Rhododendrol‐Induced Refractory Leukoderma,” Journal of Dermatology 45, no. 11 (2018): 1283–1288. 30156328 10.1111/1346-8138.14634 PMC6283075

[2] B. N. Wilson , A. Aleisa , C. Menzer , and A. M. Rossi , “Bimatoprost Drug Delivery With Fractional Laser and Microneedling for the Management of COVID‐19 Prone Positioning‐Induced Facial Atrophy and Hypopigmentation,” JAAD Case Reports 15 (2021): 26–29. 34307814 10.1016/j.jdcr.2021.07.004 PMC8280367

[3] A. S. Glaich , Z. Rahman , L. H. Goldberg , and P. M. Friedman , “Fractional Resurfacing for the Treatment of Hypopigmented Scars: A Pilot Study,” Dermatologic Surgery 33, no. 3 (2007): 289–294. 17338685 10.1111/j.1524-4725.2007.33058.x

[4] K. Beasley , J. M. Dai , P. Brown , B. Lenz , and C. M. Hivnor , “Ablative Fractional Versus Nonablative Fractional Lasers—Where Are we and How Do we Compare Differing Products?,” Current Dermatology Reports 2, no. 2 (2013): 135–143.

[5] D. M. Indramaya , M. Y. Listiawan , I. Citrashanty , et al., “The Comparison Between Microneedling and Fractional CO(2) Laser for Amniotic Membrane Stem Cell‐Conditioned Medium and Vitamin C in Photoaging Treatment,” Indian Journal of Dermatology 68, no. 4 (2023): 430–436. 37822409 10.4103/ijd.ijd_839_20 PMC10564192

[6] E. G. Baugh , O. Anagu , and K. M. Kelly , “Laser Treatment of Hypopigmentation in Scars: A Review,” Dermatologic Surgery 48, no. 2 (2022): 201–206. 34889211 10.1097/DSS.0000000000003330

[7] D. Hartmann Schatloff , C. Retamal Altbir , and F. Valenzuela , “The Role of Excimer Light in Dermatology: A Review,” Anais Brasileiros de Dermatologia 99, no. 6 (2024): 887–894. 39107199 10.1016/j.abd.2023.12.007 PMC11551234

[8] N. A. Louri , N. Dey , R. F. De Sousa , R. N. AlHasan , and M. M. Abdelhamid , “Melanocyte‐Keratinocyte Transplantation in Post‐Burn Leukoderma Scars: Preliminary Experience Using a Modified Technique,” Annals of Burns and Fire Disasters 35, no. 4 (2022): 306–314. 38680627 PMC11041886

